# Psoriasis: A STAT3-Centric View

**DOI:** 10.3390/ijms19010171

**Published:** 2018-01-06

**Authors:** Enzo Calautti, Lidia Avalle, Valeria Poli

**Affiliations:** Department of Molecular Biotechnology and Health Sciences, University of Torino, 10126 Torino, Italy; lidia.avalle@unito.it

**Keywords:** psoriasis, STAT3, Th17 cells, skin inflammation, Janus kinases, autoimmunity

## Abstract

Signal Transducer and Activator of Transcription (STAT)3 has recently emerged as a key player in the development and pathogenesis of psoriasis and psoriatic-like inflammatory conditions. Indeed, STAT3 hyperactivation has been reported in virtually every cell type involved in disease initiation and maintenance, and this factor mediates the signal of most cytokines that are involved in disease pathogenesis, including the central Interleukin (IL)-23/IL-17/IL-22 axis. Despite the recent availability of effective biological agents (monoclonal antibodies) against IL-17 and IL-23, which have radically changed the current standard of disease management, the possibility of targeting either STAT3 itself or, even better, the family of upstream activators Janus kinases (JAK1, 2, 3, and TYK2) offers additional therapeutic options. Due to the oral/topical administration modality of these small molecule drugs, their lower cost, and the reduced risk of eliciting adverse immune responses, these compounds are being actively scrutinized in clinical settings. Here, we summarize the main pathological features of psoriatic conditions that provide the rationale for targeting the JAK/STAT3 axis in disease treatment.

## 1. The Main Players in Psoriasis Pathogenesis

Psoriasis is a chronic inflammatory condition of the skin, affecting from 2% to 3% people worldwide [1,2]. Plaque psoriasis (also known as psoriasis vulgaris), is the most common clinical variant, accounting for approximately 85% of cases, and is characterized by thick erythematous plaques that are covered by silvery lamellar scales, preferentially localized on extensor surfaces, scalp, and trunk. Other less frequent clinical variants include pustular, guttate, palmoplantar, erythrodermic, and inverse psoriasis (for reviews see [3]).

Psoriasis can manifest as a relatively mild form with a limited number of lesions, or as a more severe disease, in which more than 10% of the skin surface is affected. The inflammatory events associated with psoriasis are usually not confined to the skin, as about one third of patients develop psoriatic arthritis over time; moreover, comorbid diseases of psoriasis include cardiometabolic disorder, stroke, metabolic syndrome, chronic kidney disease, mood disorders, and lymphoma, thereby accounting for an overall reduced life expectancy of patients [4,5]. Thus, psoriasis is no longer considered solely a skin disorder, but rather a systemic inflammatory disorder.

The recent identification of several autoantigens in psoriatic patients supports the notion that psoriasis is a T-cell mediated autoimmune disease, modified by genetic susceptibility and environmental stimuli (e.g., trauma, infections, and medications) [6].

Genome-wide association studies (GWAS) of psoriasis-associated genes has revealed that susceptibility to the disease involves both the adaptive and innate immune systems, and a different degree of involvement of each branch of immunity likely underlies the clinical spectrum of psoriasis (recently reviewed in [7]).

Consistent with a prominent role of immunity in psoriasis pathogenesis, susceptibility factors include several genes that are involved in antigen presentation. The strongest genetic risk factor for psoriasis susceptibility is indeed the Major Histocompatibility Complex (MHC) class I molecule Human Leukocyte Antigen (HLA-Cw*0602) [8,9]. This association is especially strong in patients with an early onset of disease [10]. Also, *Erap1* and *Erap2*, which encode for aminopeptidases involved in processing of peptides for presentation by MHC class I molecules, have been identified as susceptibility genes, with *ERAP1* variants interacting with HLA-Cw*0602 and increasing the risk for psoriasis [11,12]. MHC class I antigens are mainly involved in antigen presentation to CD8^+^ T cells, which are key effectors of the disease [13], and along with CD4^+^ T cells, represent the majority of infiltrating leukocytes that are found in skin psoriatic lesions [14,15,16]. Additional susceptibility genes are cytokines and their receptors, including *IL-12B, IL-4, IL-13, IL-23A, IL-31, IL-23R, IL-26RA*, and *IL-36RN*, many molecules that are involved in signaling by cytokines/growth factors, such as *TYK2, NFKBIA, TNFAIP3, PTEN, TRAF3, FASLG*, and the transcriptional regulators *STAT3, REL, IRF4, RUNX3, KLF4, KLF13, ILF3, MBD2, IKBKE* [12,17,18]. Several autoantigens have been identified in psoriatic patients, including keratinocyte-derived proteins, such as Keratin 17 [19,20,21] and Antimicrobial peptide (AMP) LL37 (cathelicidin) [22]. Other potential psoriasis autoantigens include melanocyte-produced A Disintegrin-like and Metalloprotease domain containing ThromboSpondin type 1 motif-Like 5 (ADAMTSL5) [23], and phospholipase A_2_ group IVD (PLA2G4D), the latter being involved in the production of non-protein lipid autoantigens in psoriatic lesions [24].

The current view on psoriasis pathogenesis is that in a genetically permissive background, epidermal antigens that are released by skin traumas or infections activate dendritic cells resident in the dermis [22,25]. Activated dendritic cells, in turn, upregulate the production of IFNα and -β, TNFα, IL-6, and IL-23 [25,26,27,28,29]. IL-6 plays a fundamental role in driving the differentiation of naive T lymphocytes into Th17 cells [30], and indeed, Th17 T lymphocytes are generally believed to play a central role in the pathogenesis of psoriasis. Among the effects of IL-6 on naive T cells is the STAT3-dependent induction of the IL-23 Receptor, which in turn, is essential to confer IL-23 responsiveness and full effector functions to Th17 cells [31]. Indeed, IL-23 is considered a “master regulator” of Th17 cell development and IL-17 production [32,33,34,35]. It is now widely accepted that IL-17-producing CD4^+^ (Th17) and CD8^+^ (Tc17) lymphocytes play a master role in psoriasis pathogenesis [16,36,37]. Additionally, resident γδ T cells, a population amplified in the dermis of psoriatic patients, have also been shown to produce IL-17, and to participate to disease pathogenesis [38,39,40].

Besides IL-17A and IL-17F, additional effector cytokines of Th17 cells are IL-6 itself, GM-CSF, TNF, IL-21, IL-22, IL-26, and IL-29 [41,42]. Together, these cytokines stimulate keratinocytes proliferation, impair their differentiation, and promote a “feed-forward” inflammatory response by activating STAT1 and STAT3, C/EBPβ, C/EBPδ and NF-κB. This leads to the upregulation of an array of pro-inflammatory genes, including chemokines attracting T cells and neutrophils and cytokines of the IL-1 family, prominently *IL-36* [43]. IL-36, in turn, further stimulates the production of IL-6, IL-23, and IL-8, and enhances IL-17 secretion by Th17 cells. This feed-forward loop is self-sustaining and ultimately causes the formation and maintenance of psoriatic plaques that are characterized by keratinocyte hyperproliferation and differentiation abnormalities, with retention of the keratinocyte nuclei in the uppermost differentiated epidermal layers (parakeratosis), and promotes the recruitment of leukocytes to the lesioned skin. In fact, both IL-17 and IL-22 stimulate the recruitment of leukocyte subsets into inflamed psoriatic lesions by inducing the production and release of chemokines such as CXCL2, CXCL3, CXCL5 and CXCL8 by keratinocytes [6,44,45]. These cytokines attract to the skin neutrophils and macrophages, which can give rise to microabscesses within the epidermal layers. IL-22 is a relatively recent addition to the increasing list of cytokines involved in psoriasis pathogenesis, playing a prominent role in the development of the psoriatic epidermal phenotype [46]. In murine models, IL-22 is mainly produced by Th17 cells. In humans, Th17 cells can produce IL-22, but the production of IL-22 without IL-17 is the hallmark of a recently identified CD4^+^ cell subset, the Th22 T cells [47,48]. Epidermal hyperproliferation is also further amplified by IL-17-induced autocrine production of IL-19 and IL-36 by keratinocytes [43]. IL-17A, IL-22, and TNF also stimulate CCL20 expression in keratinocytes, which further attracts dendritic and Th17 cells, thereby promoting a positive chemotactic inflammatory loop [44]. This feed-forward loop is further amplified by Th1 cells, which are attracted by keratinocyte-produced CXCL9, -10, and 11 upon stimulation by Th17-secreted cytokines, such as IL-26 and IL-29, and activated by DC-produced IL-12 [45]. Moreover, vascular endothelial growth factor secreted by keratinocytes stimulates angiogenesis and promotes erythema in psoriatic lesions [49].

## 2. STAT3 as a Central Player in Psoriasis Pathogenesis

As a central regulator of inflammatory and immune responses, the transcription factor STAT3 has recently emerged as a key player in the development and pathogenesis of psoriasis and psoriatic-like inflammatory conditions. Indeed, STAT3 hyperactivation has been reported in virtually every cell type involved in disease initiation and maintenance. This is consistent with the prominent role that is played by this factor in the biology of Th17 lymphocytes, and of the Th17 cytokines IL-6, IL-23, and IL-22, and with the promising results obtained with inhibitors of JAK2, one of the kinases involved in STAT3 activation downstream of many different receptors (see below).

STAT3 was initially identified as Acute Phase Response Factor (APRF), activated in IL-6-treated hepatocytes and interacting with an Acute Phase Response Element (APRE) on the promoter of several IL-6-responsive genes [50]. Since then, STAT3 is recognized as the main mediator of the functions of IL-6 in many different cell types. Its activation occurs not only downstream of all members of the IL-6 family of cytokines, but also of a great number of other cytokines, growth factors, and oncogenes, including leptin, IL-12, Interferons, IL-10, G-CSF, and Src [51], and of many cytokines that are involved in the pathogenesis of psoriasis, such as IL-21 [52,53,54], IL-22 [46,55,56], IL-23 [57,58], IL-26, and IL-29 [46,59]. Unrestrained activation of STAT3 is linked to pathologies such as neoplastic diseases and autoimmune/autoinflammatory conditions, including rheumatoid arthritis, uveitis, Multiple Sclerosis, myocarditis, and, finally, psoriasis [60,61,62]. Remarkably, germline STAT3-activating mutations have been detected in rare patients developing multi-organ autoimmunity, confirming a key role for this factor in autoimmunity [63]. Like all STAT factors, STAT3 becomes activated via phosphorylation on a conserved Tyrosine residue operated by receptor-associated JAK kinases, mainly JAK1, JAK2, and TYK2 depending on the activating stimuli [64]. STAT3 can form both homo- and heterodimers with STAT1 and binds to sequences that are very similar to the GAS sites, the typical STAT1 recognition elements [65]. One of the peculiar characteristics of STAT3 is its ability to activate different sets of genes in different cell types. This is probably achieved through specific interactions with distinct transcription factors/cofactors in different cell and promoter contexts (e.g., AP1, NF-κB, CBP/p300, NCoA-1/SRC-1, NCoR2, PCAF, GCN5, mSin3, HDACs) [66,67,68,69,70]. The final outcome of STAT3 activation in a given cellular compartment will therefore strongly depend on the cell type and on the physio-pathological context, determining a specific cytokine/growth factor milieu and coordinated activation of different repertoires of STAT3-interacting factors. Not surprisingly, STAT3 has emerged as an essential player in Th17 cells biology. Indeed, Th17 cells differentiation was abrogated in the absence of STAT3, both in vitro and in vivo [31,71], corroborated by the finding that patients with hyper immunoglobulin E syndrome (HIES), who show STAT3 inactivating mutations [72,73] display impaired Th17 differentiation that may also explain their recurrent infections [74,75]. Overexpression of a constitutively active form of STAT3, STAT3C, resulted in greatly increased numbers of IL-17 producing cells and enhanced the expression of RORγt in in vitro differentiated CD4^+^ lymphocytes [76], and a knock-in of the same STAT3C form triggered the development of autoimmune myocarditis and increased Th17 expansion [61]. Moreover, STAT3 depletion in hematopoietic stem cells or specifically in CD4^+^ lymphocytes resulted in the dramatic reduction in the number of Th17 lymphocytes [76,77]. The role of STAT3 in Th17 cell biology goes beyond it mediating IL-6-triggered differentiation, since this factor also mediates the functions of IL-23, which is required for the amplification and maintenance of Th17 responses [32,33,34,35], of IL-21, which also contributes to Th17 differentiation [78,79], and of IL-22, which mediates the cross-talk between lymphocytes and epithelial cells [46,55,59]. Not only STAT3 directly activates the *IL-17A* and *IL-17F* gene promoters [80], but also multiple other key genes that are involved in Th17 cell differentiation, activation, proliferation, and survival, such as *RORγT*, *RORα*, *BATF, IRF4, AHR, IL-6Rα, and C-MAF* [81]. Additionally, STAT3 contributes to T cell subsets expansion and homeostasis during inflammation, as it was shown to activate anti-apoptotic and pro-proliferative genes, such as *BCL2, JUN*, and *FOS*, and to inhibit T regulatory cells conversion downstream of IL-6 and IL-23 signaling [81]. Of note, STAT3 appears to be also required for the expansion and function of γδ T cells, which contribute to psoriasis pathogenesis by dermal production of IL-17 [38,39,40]. Notably, the IL-7-induced expansion of IL-17-producing γδ T cells in B16-F10 melanoma is abolished by STAT3 inhibition [82], and the inhibition of IL-23-induced expansion of antigen-induced γδ T cell subsets in tubercolosis correlates with dicreased expression and phosphorylation of STAT3 [83].

**Figure 1:**
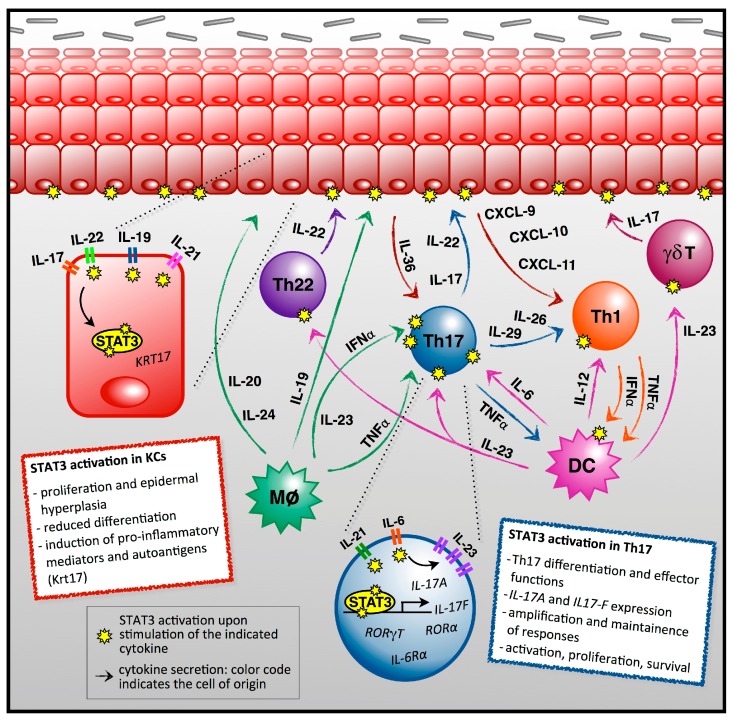
The scheme depicts the main cell types involved in psoriasis pathogenesis, the cytokines/chemokines produced and their target cells. The arrows indicate cytokines that are produced by the different cell types, and the cells where these mainly exert their effector functions. The yellow stars represent events of cytokine-induced STAT3 activation. In the blow-ups, indicated by the dotted lines, the two main cell types involved, Th17 CD4^+^ T lymphocytes, and keratinocytes, along with details of STAT3-mediated functions and a description of their main pathogenetic features are represented. See text for details and references.

Many lines of evidence intimately link STAT3 aberrant activity with the development and progression of psoriasis, starting from the observation of high levels of activated STAT3 in the skin of psoriatic patients (Figure 1, Table 1, [62]). As already mentioned, this factor is essential to Th17 differentiation in a number of ways, and it mediates the signalling of most cytokines that are involved in Th17 (and Th22) cells differentiation and effector functions. Additionally, STAT3 plays a central role in the pathological responses of keratinocytes to inflammatory T cell cytokines. Indeed, STAT3-mediated IL-21 induction by IL-6 in naive T cells triggers a positive loop involving STAT3, enhancing IL-21 production, induction of IL-23 receptor, and finally, IL-17 expression [31]. IL-21 levels in the skin are associated to psoriasis severity [84], and this cytokine acts on keratinocytes by inducing proliferation and epidermal hyperplasia signalling via STAT3 [85]. On the other hand, also IL-22 triggers reduced differentiation, increased proliferation, and acanthosis in psoriatic keratinocytes/epidermis via STAT3 activation [46,86,87]. Other cytokines of the IL-20 subfamily, and in particular, IL-19, signalling via STAT3, are involved in amplifying the IL-23/IL-17 axis and the induction of pro-inflammatory mediators, wound healing factors and inhibitors of differentiation in keratinocytes [59,88]. STAT3 has also been implicated in inducing the expression of Keratin 17 [89,90,91], a proposed autoantigen in psoriasis, downstream of IL-22, IL-17, and other cytokines [46,92,93]. Of note, we have observed increased Keratin 17 levels in knock-in mice expressing constitutively active STAT3, which develop epidermal hyperplasia and display enhanced clonogenic potential of keratinocytes [94]. STAT3 is also involved in mediating the synergy between TNFα and IL-22/IL-24 in skin inflammation [87,95]. Additionally, IL-6-activated STAT3 is implicated in mediating the escape of T effector cells from the control of T Regulatory cells [96], and in mediating the loss of suppressive power in Treg cells downstream of IL-6, IL-21, and IL-23 [54]. Finally, several GWA studies detected correlations between psoriasis and alternative forms of the STAT3 locus [12,17,18]. Confirming the central role that is played by STAT3 in psoriasis pathogenesis, the groups of Di Giovanni and Sano have demonstrated that *Keratin5.Stat3C* transgenic mice, overexpressing constitutively active STAT3 in keratinocytes, develop a skin phenotype closely resembling psoriasis in response to wounding or to TPA treatment. The development of psoriatic lesions requires the activity of T lymphocytes, is dependent on IL-23 (and to a lesser extent on IL-17), and can be prevented by the topical application of a STAT3 inhibitor [62,97,98]. Strikingly, the same inhibitor could improve psoriatic lesions in six out of eight patients that were treated as part of a non-randomized trial.

## 3. Biological Therapeutic Strategies (Anti-IL-17, -23, -22) and JAK Inhibitors

The discovery of the central role of the IL-23/Th17 signaling axis in psoriasis pathogenesis has led to the development of biologics such as IL-23- or IL-17-blocking monoclonal antibodies, which represent perhaps the most effective line of treatment of psoriasis known to date [6,99,100]. These compounds have proven superior in the management of psoriatic disease to TNF antagonists, the efficacy of which likely depends on indirect inhibition of the IL-23/IL-17 pathway. The JAK/STAT3 dependent cytokine IL-23 is a fundamental mediator of psoriasis, since it stimulates Th17 cells to produce IL-17, which is another key molecule in psoriasis pathogenesis [57,58]. Although IL-17 does not rely directly on JAK/STAT3 signaling, blockade of upstream IL-23 via JAK inhibitors leads to an overall decrease in IL-17 levels [101,102]. Moreover, IL-6, IL-21, IL-22, IL-26, and IL-29, which participate to the amplification and maintenance of the inflammatory loop in psoriasis, all signal via STAT3 [46,52,53,55,56,59,103]. Therefore, the development and clinical testing of small-molecule inhibitors targeted to upstream JAK enzymes in psoriasis management is an area of active investigation in the field. The efficacy and safety of these compounds in the management of psoriasis, as well as other inflammatory skin disorders, is clearly emerging in clinical settings, as recently reviewed in detail [104,105,106]. We briefly summarize here some particularly promising results that were obtained with JAK inhibitors in the treatment of psoriasis.

Tofacitinib, an oral JAK1 and-3 inhibitor that is already approved for use in rheumatoid arthritis (RA), [107,108,109], is currently under evaluation for the treatment of both plaque psoriasis [110] and psoriatic arthritis [111,112,113]. In a phase 3, randomized clinical trial, the 10 mg twice daily dose of tofacitinib was not inferior to 50 mg twice weekly of subcutaneously-administered etanercept (TNFα inhibitor) over a period of 12 weeks in patients with moderate-to-severe plaque psoriasis, with a similar safety profile between the two treatment regimens [114]. As previously reported in clinical studies on RA, tofacitinib-treated patients showed, as side effects, increases in both LDL and HDL cholesterol and mild increases in creatine phosphokinase, the clinical significance of which are presently unclear. Changes in hematological parameters, such as lymphocyte count and haemoglobin concentration, which could potentially result from the suppression of JAK signaling in lymphopoiesis and haematopoiesis, respectively, were modest. In other two randomized phase 3 trials that were carried out over a 16 weeks period, oral tofacitinib at 5 or 10 mg doses twice daily demonstrated also a significant efficacy versus the placebo group, with nasopharyngitis emerging as the most frequent adverse reaction, while a minority of patients (7 out of 1486) developed herpes zoster complications [115]. Because of the chronic nature of the disease, however, it will be essential to confirm the safety profiles of the compound over longer periods of time. Interestingly, tofacitinib proved to be effective in improving pruritus and quality of life in moderate-to severe plaque psoriasis patients in two recent phase 3 studies carried out over 52-weeks [116]. Another recent study confirmed the positive effects of tofacitinib on itch relieve, indicating significant improvements as soon as two days after the beginning of treatment [117]. Other oral JAK inhibitors are currently under development and/or at early stages of clinical testing.

Ruxolitinib is a JAK1 and -2 inhibitor that is currently under investigation for use in the form of topically applied cream. In a phase 2 clinical trial conducted in patients with limited psoriasis (less than 20% of body surface are affected), ruxolitinib proved to be safe, well tolerated, and exhibited significant clinical activity in the topical treatment of psoriasis. Although the study was limited by the relatively short duration and small sample size, it indicated that ruxolitinib might represent a promising drug for topical treatment of psoriasis [118].

Thus, JAK inhibitors may offer an alternative modality of treatment with several potential advantages over biologic medications. For instance, biologics must be administered by injection, whereas small molecules can be administered orally and/or topically. Secondly, small molecules are relatively inexpensive as compared to biologic agents, and lack the immunogenic properties of human- or humanized monoclonal antibodies. Therefore, it is tempting to speculate that targeting the JAK/STAT pathway, rather than simply interfering with an individual (although essential) cytokine, might elicit favorable therapeutic responses in patients refractory to biologics, by intercepting the self-sustaining hyperproliferative inflammatory loop at multiple levels, in which several JAK/STAT3-dependent cytokines and/or target genes play important roles in disease amplification and maintenance, including the expression of putative psoriatic autoantigens (e.g., Krt17) in epidermal keratinocytes. In support of this concept, it has been recently reported that psoriatic keratinocytes overexpress JAK1 and -3, making them ideal targets for topical treatment with broad-specific JAK inhibitors [119].

In summary, all the experimental and clinical data support the central role of STAT3, and of its activating JAK kinases, in the onset, development, and progression of psoriasis and open up novel therapeutic opportunities for the management not only of this disease, but possibly also other autoinflammatory and autoimmune diseases affecting the skin and other districts.

## Figures and Tables

**Table 1:** Biological functions of STAT3 in the main cell types involved in psoriasis pathogenesis. The table reports the cell type, the cytokines shown to activate STAT3 in different contexts, and the main biological functions exerted by this factor in the specific cell type considered. The main references are shown.

Cell Type	Cytokine	Effect	Refs.
Th17	IL-6	differentiation, induction of IL-23 receptor	[30,31]
	IL-23	amplification and maintainance	[78]
	IL-21	differentiation	[79]
	IL-22	cross-talk lymphocytes-epithelial cells	[46]
	IL6, IL-23	activates *IL-17A* and *IL-17F*, *RORγT, RORα, BATF, IRF4, AHR, IL-6Rα* and *C-MAF*	[81]
Keratinocyte	IL-21	proliferation and epidermal hyperplasia	[85]
	IL-22	proliferation, reduced differentiation and acanthosis	[46,86,87]
	IL-19	amplification of IL-23/IL-17 axis, and induction of pro-inflammatory mediators	[59,88]
	IL-22, IL-17	*KRT17* induction	[46,89,90,91,92,93]
γδ T cells	IL-7	expansion	[82]
	IL-23	expansion	[83]

## Abbreviations

GWAS	Genome-wide association studies
RA	Rheumatoid arthritis
HIES	Hyper immunoglobulin E syndrome
